# Automated Synthesis of Radiopharmaceuticals: Technology, Applications, and Regulatory Perspectives

**DOI:** 10.1007/s13139-025-00956-8

**Published:** 2025-10-16

**Authors:** Nare Ko, Sang Ju Lee, Seung Jun Oh

**Affiliations:** 1 Department of Nuclear Medicine, Asan Medical Center, University of Ulsan College of Medicine, Seoul, Republic of Korea; 2 Biomedical Research Center, Asan Institute for Life Sciences, Seoul, Republic of Korea

**Keywords:** Radiopharmaceuticals automation, Purification, Radioisotope, Radiopharmaceuticals, GMP

## Abstract

The automatic synthesis of radiopharmaceuticals has a long history, from [^18^F]FDG to the most recent metallic radioisotope-labeled radiopharmaceuticals. Initially, the development of the automatic synthesis process focused on radioisotope labeling and high yields, as well as other chemical processes such as HPLC purification. The initial focus of the development of the [^18^F]FDG synthesis automatic chemistry module was to prevent radiation exposure and to develop a reproducible process. The synthesis procedure and purification methods were continuously developed to reduce preparation time and simplify purification, for example by using solid-phase extraction, to achieve high radiochemical yields and purity. Recently, there have been automatic chemistry modules optimized for metallic radioisotope labeling, and these machines have demonstrated very high radiochemical yields and short synthesis times. Compared with previous developments, the recent automatic preparation process includes simple chemical synthesis to satisfy GMP regulations because all radiopharmaceutical processes are trackable and reproducible. In this review paper, we describe the development process for each radioisotope labeling for radiopharmaceuticals, as well as the regulatory aspects that satisfy recent GMP regulations.

## Introduction

Over the past several decades, nuclear medicine has undergone a profound transformation, driven by the growing need for precise, efficient and safe diagnostic and therapeutic approaches. Radiopharmaceuticals, which are compounds labeled with radionuclides, enable precise imaging and treatment via positron emission tomography (PET) and single photon emission computed tomography (SPECT). Advances in radiotracer design and synthesis have substantially improved the sensitivity of diagnosis, the specificity of therapy, and the clinical utility of radiopharmaceuticals across a broad spectrum of medical applications [1–3].

Initially, radiotracer synthesis was performed manually inside lead-shielded glove boxes or hot cells. While this method facilitated the initial optimization of chemical reactions, purification steps and quality control, it was labor-intensive and inconsistent in terms of radiochemical yield and purity. Furthermore, it posed a significant radiation risk to operators [4, 5]. As the demand for reproducible, Good Manufacturing Practice (GMP)-compliant and time-sensitive radiopharmaceutical production increased in a clinical setting, the limitations of manual synthesis became evident. This led to the development of automated synthesis systems that offer enhanced safety, operational efficiency and scalability [6, 7]. 

The development of the radiopharmaceutical production automation process started with PET radiopharmaceuticals because of the high level of radioactivity required for production and the positron’s high energy. The growing demand for the rapid, remote and reliable production of PET radiopharmaceuticals has led to the development of various automated synthesis modules. This became a particular concern with the introduction of short-lived positron-emitting radionuclides, such as carbon-11 (^¹¹^C) and oxygen-15 (^¹⁵^O), as synthesis and quality control must be completed within very short timeframes [8–11]. Consequently, the first automation systems emerged in the 1980s and 1990s to meet these stringent time constraints [14–16]. The successful implementation of these systems in PET radiochemistry enabled the process to be adapted for the synthesis of a broader range of radiopharmaceuticals, expanding its utility to include both diagnostic and therapeutic applications [12].

Advancements in automation technology have progressed alongside developments in radiochemistry. Contemporary synthesis platforms incorporate features such as single-use sterile cassettes, programmable logic controllers, in-line quality control systems, and emerging microfluidic technologies [13, 14]. Together, these innovations improve reproducibility, minimize radiation exposure and support adherence to GMP standards, thereby facilitating the reliable production of radiopharmaceuticals in clinical and research settings [15, 16].

**Fig. 1:**
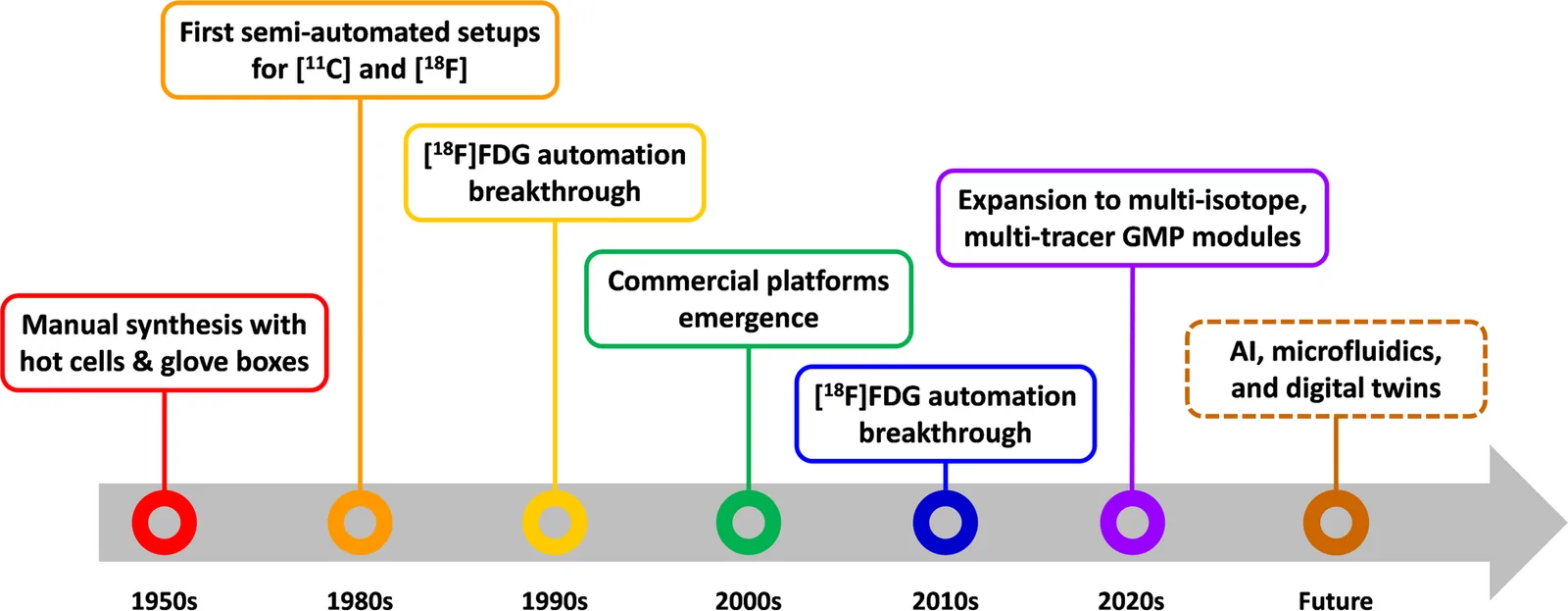
Timeline highlighting key milestones in the evolution of radiopharmaceutical synthesis from manual processes to modern cassette-based automation and next-generation microfluidic and AI-assisted systems

This review provides a thorough overview of the evolution, current practices and future prospects of the automated synthesis of radiopharmaceuticals. It emphasizes significant advancements in system architecture, isotope-specific processes, regulatory considerations, and emerging technological innovations. By synthesizing these developments, the review aims to provide a clear framework for understanding the current landscape and illuminate pathways for future innovation in automated radiopharmaceutical production. A summary of key historical milestones in radiopharmaceutical synthesis is illustrated in Fig. 1.

## Importance of Automated Synthesis

The routine clinical production of radiopharmaceuticals, particularly those involving high-radioactivity therapeutic radionuclides such as [^177^Lu] or positron emitters with high-energy gamma emissions such as [^18^F]fluoride, requires robust automated synthesis platforms. The transition from manual to automated procedures is driven by three fundamental imperatives: radiation safety, consistent manufacturing under GMP and facilitating clinical translation and broader access to novel agents.

First, automation is a critical tool of adhering to the ‘As Low As Reasonably Achievable’ principle of radiation safety [17, 18]. Manual synthesis of multi-gigabecquerel batches of radiopharmaceuticals exposes operators to significant radiation doses. Automated synthesis modules housed within shielded hot cells and operated remotely minimize this exposure, ensuring a safer working environment for laboratory personnel.

Second, automation is essential for achieving the high level of reproducibility and quality required for GMP-compliant manufacturing [19]. Automated systems execute pre-validated synthesis protocols with precise control over reaction parameters such as temperature, timing and reagent volumes. This minimizes the variability between operators inherent in manual preparations. This consistency is crucial for ensuring that each batch of a radiopharmaceutical meets the stringent quality control specifications required for identity, purity and sterility for clinical use [20]. Using optimized and sterile, disposable cassette systems also improves product quality by preventing cross-contamination between production runs.

**Fig. 2:**
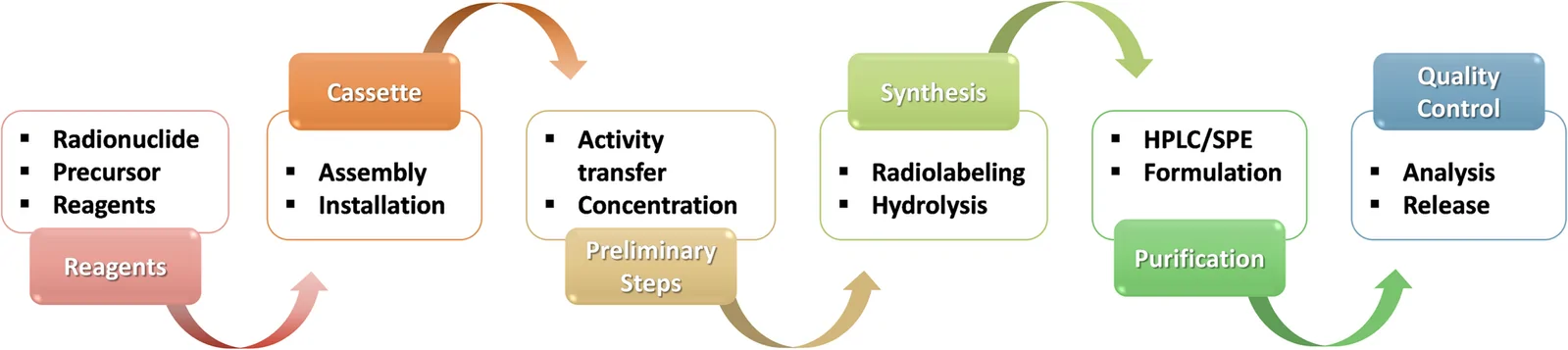
Simplified schematic of a typical cassette-based automated synthesis process

Ultimately, robust and reproducible automated synthesis methods are essential for the clinical translation and widespread adoption of novel radiopharmaceuticals. Manual production methods can present significant technological and economic challenges that restrict access to complex agents such as [^89^Zr]-labeled antibodies to a few specialized centers [21]. Automated platforms provide a standardized, validated and user-friendly production pathway, thereby lowering the barrier for multicenter clinical trials and routine clinical implementation and improving patient access to state-of-the-art diagnostic and therapeutic agents [22]. The typical configuration and workflow of a cassette-based automated synthesis platform are depicted in Fig. 2.

## Automated Synthesis of Radiopharmaceuticals

Automated synthesis systems must be able to accommodate a wide variety of radiopharmaceuticals, which are defined by factors such as the type of radionuclide, its physical half-life, chemical characteristics and clinical application. Radiopharmaceuticals are broadly classified based on their isotopic origin into two main groups: organic (non-metallic) and metallic radionuclides. The evolution of automation has been influenced by the distinct technical requirements associated with each class, including differences in labeling chemistry, purification strategies, and regulatory constraints. Table 1 provides a summary of selected commercial automated synthesis platforms, the radionuclides they support, and their primary clinical and research applications.

**Table 1:** Overview of selected commercial automated synthesis platforms and their applications

Platforms	Radionuclides	Key features	Applications	References
Eckert & Ziegler Modular-Lab	[^68^Ga], [^177^Lu]	Modular design; user-programmable for R&D and production	[^68^Ga]Ga-FAPI-46, [^68^Ga]Ga-ABY-025, [^177^Lu]Lu-DOTATATE, [^177^Lu]Lu-FAPI-04/64	[58, 59, 72, 74]
Elysia-Raytest GAIA	[^68^Ga]	Cassette-based; optimized for peptides; intuitive operation	[^68^Ga]Ga-EMP100, [^68^Ga]Ga-NODAGA-Lys40-Exendin-4	[58, 60]
GE FASTlab/ TRACERlab	[^18^F], [^68^Ga]	Cassette-based; optimized for routine clinical production	[^18^F]FDG, [^18^F]FET, [^18^F]FDOPA, [^18^F]Fcholine, [^18^F]FSPG, [^68^Ga]Ga-FAPI-46	[26, 28, 30–32]
iPHASE Multisyn	[^89^Zr], [^11^C], [^177^Lu]	Cassette-based; adapted for biologics and therapeutic agents	[^89^Zr]Zr-DFOSq-Durvalumab, [^11^C]C-FMDS, [^89^Zr]Zr-DFOSq-bisPh(PSMA)/TATE, [^177^Lu]Lu-PSMA-617	[22, 41, 69, 71]
Scintomics GMBH/GRP	[^18^F], [^68^Ga]	Suitable for both GMP manufacturing and research; modular	[^18^F]TRACK, [^68^Ga]Ga-DOTATATE, [^18^F]FDG	[36, 53]
Trasis (mini) All-In-One	[^18^F], [^68^Ga], [^177^Lu]	Highly flexible; user-programmable; multi-tracer capability	[^18^F]SynVesT-1, [^18^F]FDOPA, [^18^F]FP-TMP, [^18^F]FET, [^18^F]Fallypride, [^18^F]SiTATE, [^18^F]Ga-rhPSMA-7/−7.3, [^68^Ga]Ga-FAPI-46, [^68^Ga]PSMA-HBED-CC, [^177^Lu]PSMA-I&T	[19, 29, 35, 57,73 38, ]

### Organic Radionuclides

#### Fluorine-18 ([^18^F])

Fluorine-18 (^18^F) is the most widely used radionuclide in positron emission tomography (PET) due to its favorable physical and chemical properties. With a half-life of around 110 min, it strikes an optimal balance between spatial resolution and logistical feasibility, allowing for both centralized production and regional distribution. These advantages have led to the widespread clinical adoption of fluorine-18-labeled tracers in oncology, neurology and cardiology.

The automation of [^¹⁸^F]FDG production was a major turning point for radiopharmaceuticals, significantly advancing the clinical adoption of PET imaging. Previously synthesized manually under high-radiation conditions, [^¹⁸^F]FDG is the most well-established radiopharmaceutical and sets the standard for automated, cassette-based, GMP-compliant synthesis. Commercial platforms such as the GE FASTlab, the Trasis All-In-One and the Scintomics GRP have been developed to meet the demand for high-throughput, reproducible production. These systems consequently offer standardized production protocols, enhanced safety and improved reproducibility across production sites [23–25].

There were many different types of automatic synthesis chemistry module available for [^18^F]FDG production. The GE FASTlab enables the efficient, one-touch synthesis of [^18^F]FDG [26, 27] as well as other clinically validated tracers, including [^18^F]FDOPA [28, 29], [^18^F] choline [30], (S)−4-(3–18 F-Fluoropropyl)-L-glutamic acid ([^18^F]FSPG) [31, 32]. The Trasis All-In-One platform offers flexible multi-tracer capability with programmable synthesis protocols and integrated quality control modules [33–35]. Scintomics GRP supports both GMP manufacturing and research-oriented operations, making it suitable for academic and hybrid environments [36–38]. Using sterile disposable cassette kits across these platforms reduces the risk of cross-contamination, enhances reproducibility and facilitates compliance with international GMP standards. Consequently, ^¹⁸^F radiochemistry remains the reference model for automated radiopharmaceutical production, supporting both routine clinical applications and the translation of novel tracers into clinical trials.

#### Carbon-11 ([^11^C])

Carbon-11 (^11^C) is a key element in most pharmaceutical compounds. This intrinsic property enables it to be incorporated into molecular structures without significantly altering their chemical integrity or biological activity [39]. Consequently, [^11^C]-radiolabeling preserves the native pharmacokinetics of precursor compounds, thereby enhancing the translational relevance of preclinical findings in animal models and human studies.

Despite the significant advantages of [^11^C], its short physical half-life of 20.4 min means that radiopharmaceutical synthesis, quality control, and patient administration must be carried out quickly, usually within a single on-site facility. Typical radiochemical reactions for [^11^C]-labeling strategies include [^11^C]methylation [40], [^11^C]carboxylation [41], [^11^C]cyanation [42], and [^11^C]carbonylation [43]. Clinically relevant and widely studied radiotracers include [^11^C]methionine as a cancer biomarker [44], [^11^C]raclopride as a biomarker of Parkinson’s disease [45], and [^11^C]Pittsburgh compound-B as a biomarker of Alzheimer’s disease [46].

Although the diagnostic value of [^11^C]-labeled radiopharmaceuticals is well recognized, they have not been widely adopted in clinical practice. This is primarily due to the requirement for on-site cyclotron facilities, specialized infrastructure, and trained radiochemistry personnel [47]. Overcoming these logistical and technical barriers would significantly increase the clinical potential of [^11^C], which remains a valuable tool in translational research. In particular, [^11^C] continues to contribute meaningfully to advancements in neuroimaging, receptor mapping, and molecular pharmacology. Carbon-11 ([^11^C]) is the foundational element of most pharmaceutical compounds.

### Metallic Radionuclides

#### Gallium-68 ([^68^Ga])

Gallium-68 ([^68^Ga]) is a positron-emitting radionuclide produced by generators with a physical half-life of approximately 68 minutes. As it can be easily obtained from ^68^Ge/^68^Ga generators rather than a cyclotron, [^68^Ga] offers a cost-effective, decentralized and on-demand alternative to cyclotron-produced [^18^F]-tracers [48]. This generator-based approach has markedly increased PET accessibility in clinical and research settings [49, 50].

The rising clinical demand for PET tracers, coupled with the need to mitigate operator exposure to the high positron emission energy of [^68^Ga], has driven the automation of [^68^Ga] radiochemistry synthesis processes. Standard automated synthesis processes commence with generator elution, followed by fractionation or cation-exchange purification to isolate [^68^Ga]Ga^3+^ in an acidic medium [51]. The purified eluate is then buffered and reacted with macrocyclic ligands such as DOTA, HBED, or NOTA under controlled temperature and pH to achieve complexation yields of over 95%. Representative tracers produced via this route include [^68^Ga]Ga-DOTATATE, [^68^Ga]Ga-DOTATOC, [^68^Ga]Ga-PSMA-11, and [^68^Ga]Ga-FAPI-46 [52–57].

Emerging [^68^Ga] radiotracers further illustrate the versatility of this chemistry. Automated syntheses of HER2 targeting [^68^Ga]Ga-ABY-025 on the Modular-Lab PharmTracer (Eckert & Ziegler) [58], c-MET targeting [^68^Ga]Ga-EMP100 on the GAIA synthesis module (Elysia Raytest) [59], and GLP1 receptor-targeting [^68^Ga]Ga-NODAGA-Lys^40^-Exendin-4 on the Modular-Lab Standard (Eckert & Ziegler) [60] routinely achieve high radiochemical purity and molar activities suitable for human PET applications.

#### Zirconium-89 ([^89^Zr])

Zirconium-89 ([^89^Zr]) is a positron-emitting radionuclide produced by the ^89^Y(p, n)^89^Zr nuclear reaction using natural yttrium-89 targets. With a physical half-life of 78.4 h (3.3 days), [^89^Zr] is substantially longer-lived than conventional PET isotopes such as [^18^F] (109.8 min) and [^68^Ga] (67.7 min). This makes zirconium-89 particularly well suited for labeling biomolecules with slow pharmacokinetics, including monoclonal antibodies, peptides, and cell-based therapeutics. This extended half-life enables comprehensive whole-body biodistribution, patient stratification, and accurate dosimetry for both diagnostic and theranostic applications [61–66].

The preferred chelator for [^89^Zr] is desferrioxamine B (DFO), which forms stable complexes with Zr^4+^ via its hydroxamate groups. DFO is typically conjugated to targeting biomolecules using reactive linkers, such as maleimide, NHS ester, or isothiocyanate moieties, which enable covalent attachment to lysine or cysteine residues under mild basic conditions (pH 8.5–9.5) [67]. Radiolabeling of antibody-based [^89^Zr] tracers requires careful control of pH (6.8–7.5) and moderate temperatures (37–40 °C) to maintain protein stability while achieving high labeling efficiency and specific activity.

The growing number of clinical trials incorporating [^89^Zr]-labeled antibodies for patient selection and monitoring of therapeutic response has driven the development of automated synthesis platforms designed specifically for this radioisotope. Schomäcker et al., highlights the translational development process of [^89^Zr]Zr-PSMA-DFO for delayed PET imaging of recurrent prostate cancer, emphasizing how interdisciplinary cooperation enables patient-centered radiopharmaceutical innovation from bench to bedside [20]. Wichmann et al., reports on the development and clinical process validation of a fully automated cassette-based method to synthesize [^89^Zr]Zr-DFOSq-Durvalumab for immuno-PET of PD-L1 expression, enabling consistent multi-center production for a clinical trial using iPHASE MultiSyn platform [22]. Noor et al., describes fully automated process for the synthesis of [^89^Zr]Zr-DFOSq-bisPh(PSMA)/TATE in high radiochemical yields with high radiochemical purity using iPHASE Multisyn module [68].

#### Lutetium-177 ([^177^Lu])

Lutetium-177 ([^177^Lu]) is a therapeutic radionuclide with a half-life of approximately 6.7 days, making it highly suitable for targeted radionuclide therapy (TRT). Its clinical success, exemplified by FDA-approved drugs Lutathera^®^ ([^177^Lu]Lu-DOTATATE) and Pluvicto^®^ ([^177^Lu]Lu-PSMA-617), has established [^177^Lu]-based radiopharmaceuticals as transformative treatments for neuroendocrine tumors and prostate cancer, respectively. This has driven significant industry investment to expand production and develop next-generation agents for broader oncological use [69]. 

The successful implementation of automated synthesis has been demonstrated for several clinically significant [^177^Lu]-labeled radiopharmaceuticals across various commercial platforms. For instance, Wichmann et al. described the fully automated, cassette-based clinical production of [^177^Lu]Lu-PSMA-617 on the iPHASE MultiSyn module. This process demonstrated high yields and purity (> 95%) that met all GMP quality criteria [70]. The long-term reliability of such systems was highlighted by Aslani et al., who detailed one year of consistent GMP production experience for [^177^Lu]Lu-DOTATATE using the Eckert & Ziegler Modular-Lab PharmTracer^®^ system, which yielded consistently high radiochemical purities (> 99%) and underscored the benefits of automation for safe, reproducible therapy [71]. Likewise, Sørensen et al. established robust, GMP-compliant protocols for [^177^Lu]Lu-PSMA-I&T on the Trasis mini All-In-One module, facilitating integrated diagnostic and therapeutic radiolabeling [72]. Moreover, the application of this technology has been extended to novel agents, with Eryilmaz and Kilbas achieving the first fully automated synthesis of [^177^Lu]Lu-FAPI derivatives on the Modular Lab Eazy platform, reporting high yields (86%–88%) and validated stability suitable for clinical theranostic use [73]. 

## Current Limitations and Unmet Needs

Automated synthesis platforms have become essential for modern clinical radiopharmaceutical production. They provide a robust foundation that enhances operator safety, ensures consistent production and supports strict compliance with Good Manufacturing Practice (GMP). However, the growing demand for complex biomolecules and personalized theragnostic has revealed significant limitations in the capabilities of current systems. A primary challenge lies in the lack of operational flexibility. Innovation is often constrained by rigid, vendor-specific hardware configurations and dependence on proprietary single-use consumables and cassettes. Furthermore, the full integration of complex post-processing steps, such as the purification of generator eluate or the removal of metallic impurities, into closed, single-pass processes remains technically challenging, particularly for emerging radioisotopes and novel ligands. Quality control also remains a critical bottleneck as it still relies heavily on manual sampling and offline testing. This combination of technical, operational and economic constraints limits the realization of fully integrated synthesis, adds to operational costs and hinders the scalability and rapid clinical translation of next-generation radiopharmaceuticals.

Recently, the concept of data integrity has become mandatory for all types of automated chemistry modules under GMP regulations. This concept involves recording, reviewing and locking all kinds of data, such as login to the control system, temperature, pressure and radioactivity, under an internally approved process to satisfy GMP regulations. To comply with data integrity regulations, companies that produce automatic chemistry modules have developed new operation software and launched it.

## Future Directions and Innovations

The future of automated radiopharmaceutical production is set to overcome current challenges by integrating advanced, flexible technologies. Microfluidic technology such as ‘lab-on-a-chip’ systems are among the most significant of these advancements [74–76]. These platforms are set to dramatically reduce reagent volumes, accelerate reaction kinetics and boost radiochemical yields while minimizing operator radiation exposure. The convergence of microfluidics with compact generator technologies and AI-driven process monitoring is expected to catalyze a paradigm shift from centralized production to a decentralized, ‘dose-on-demand’ manufacturing model. This approach would enable the preparation of patient-specific doses, thereby expanding access to personalized radiopharmaceuticals, particularly for facilities lacking cyclotron infrastructure [77, 78].

**Fig. 3:**
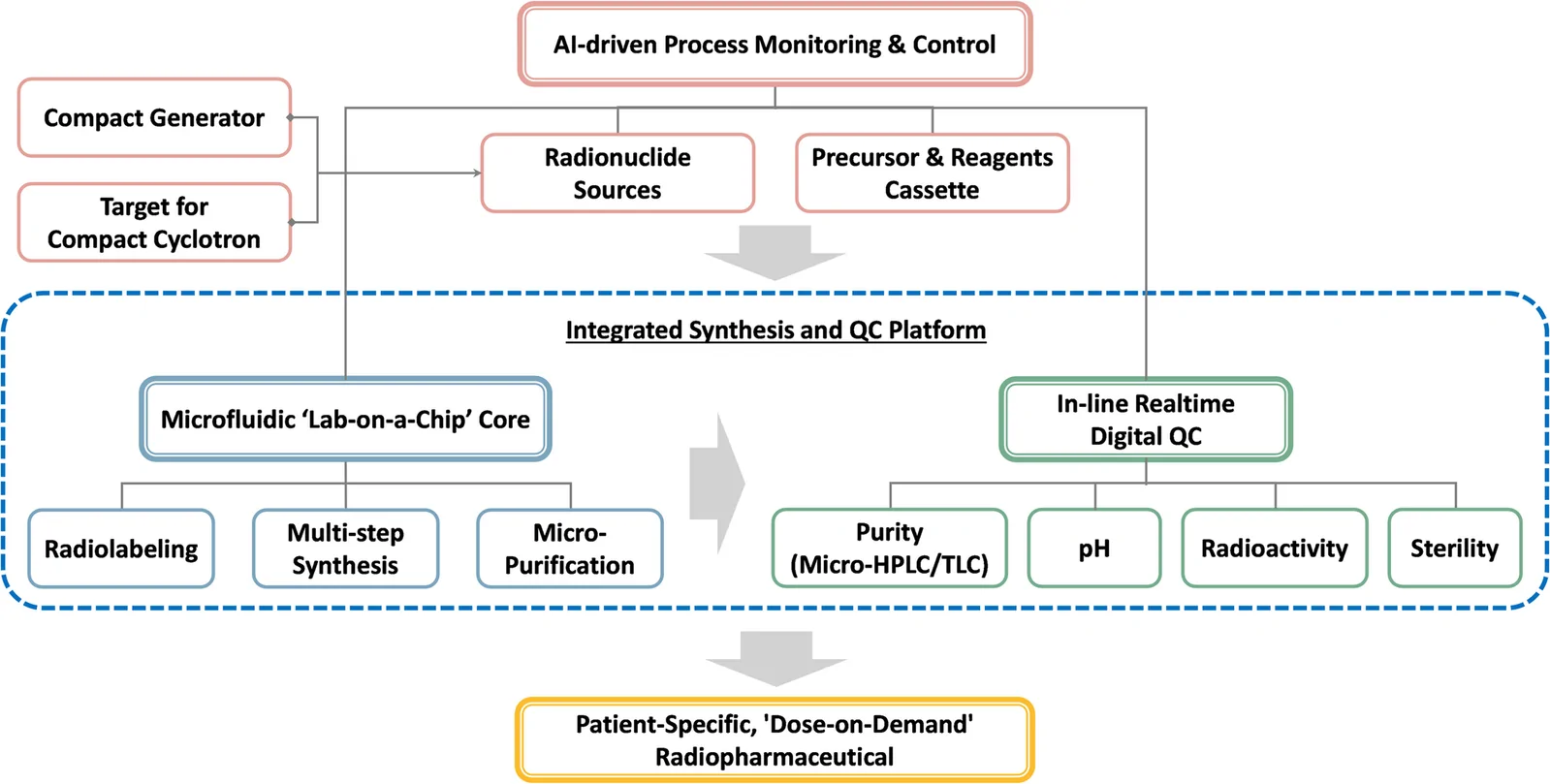
Conceptual schematic of a future fully integrated radiopharmaceutical synthesis platform

The long-term vision is to develop fully integrated, multi-isotope systems that combine radionuclide production, multi-step synthesis, purification and in-line, real-time digital quality control within a single, self-contained unit. This would streamline operations and minimize user intervention. A conceptual schematic of such a next-generation, intelligent synthesis platform is presented in Fig. 3.

As regulatory expectations evolve, it will be crucial to lower barriers to technology adoption by achieving global harmonization of GMP standards and creating validated software libraries. Continued collaboration between industry, academia and regulatory organizations is essential to ensure that automated radiopharmaceutical production remains safe and efficient and is capable of meeting the growing clinical demand for precision nuclear medicine. Addressing the current challenges in automation is vital to realize the full potential of this field. Greater flexibility and end-to-end integration will optimize the production of theranostic agents and democratize access to novel radiopharmaceuticals. This will accelerate the discovery, validation and clinical translation of next-generation imaging and therapeutic agents, bringing the promise of personalized medicine from bench to bedside more rapidly.

## Data Availability

Not applicable.
